# Sex Differences in Physical Activity and Its Association With Cardiac Death and All-Cause Mortality in Patients With Implantable Cardioverter-Defibrillators

**DOI:** 10.3389/fcvm.2020.588622

**Published:** 2020-12-14

**Authors:** Xiaoyao Li, Xiaodi Xue, Xuerong Sun, Shuang Zhao, Keping Chen, Wei Hua, Yangang Su, Jiefu Yang, Zhaoguang Liang, Wei Xu, Zeyi Li, Shu Zhang

**Affiliations:** ^1^Arrhythmia Center, State Key Laboratory of Cardiovascular Disease, Fuwai Hospital, National Center for Cardiovascular Diseases, Chinese Academy of Medical Sciences and Peking Union Medical College, Beijing, China; ^2^Department of Cardiology, Shanghai Institute of Cardiovascular Diseases, Zhongshan Hospital, Fudan University, Shanghai, China; ^3^Department of Cardiology, Beijing Hospital, Beijing, China; ^4^Department of Cardiology, First Affiliated Hospital of Harbin Medical University, Harbin, China; ^5^Department of Cardiology, Nanjing Drum Tower Hospital, Nanjing, China

**Keywords:** physical activity, sex differences, cardiac death, all-cause mortality, implantable cadioverter defibrillators

## Abstract

**Objective:** To clarify the impact of sex on physical activity (PA) levels among patients with implantable cardioverter-defibrillators/cardiac resynchronization therapy defibrillators (ICD/CRT-D) and its association with cardiac death and all-cause mortality.

**Methods:** Overall, data of 820 patients with ICD/CRT-D from the SUMMIT registry were retrospectively analyzed. Baseline PA from 30 to 60 days after device implantation was measured using Biotronik accelerometer sensors. The primary and secondary endpoints were cardiac death and all-cause mortality, respectively.

**Results:** Baseline PA levels were significantly higher in male patients than in female patients (11.40 ± 5.83% vs. 9.93 ± 5.49%, *P* = 0.001). Males had higher predictive PA cut-off values for cardiac death (11.16 vs. 7.15%) and all-cause mortality (11.33 vs. 7.17%). During the median follow-up time of 75.7 ± 29.1 months, patients with baseline PA<cut-off values had higher cumulative incidence of cardiac death and all-cause mortality in both males and females. At a PA level between the cut-off values of males and females, males had a higher risk of cardiac death (hazard ratio = 4.952; 95%CI = 1.055-23.245, *P* = 0.043) and all-cause mortality (hazard ratio = 2.432; 95%CI = 1.095-5.402, *P* = 0.029).

**Conclusions:** Males had higher predictive PA cut-off values for cardiac death and all-cause mortality in patients with ICD/CRT-D. Sex should be considered as an important contributing factor when deciding for PA targets.

## Introduction

Implantable cardioverter-defibrillators (ICD) and cardiac resynchronization therapy defibrillators (CRT-D) have become the standard modalities for the prevention of sudden cardiac death and management of some patients with heart failure (HF) (1, 2). It is important to identify patients at high risk for cardiac death and all-cause mortality after ICD/CRT-D implantation to improve their clinical outcomes (3–6).

Physical activity (PA) is a quantitative index reflecting individual functional status.

A number of studies have indicated that PA is significantly associated with clinical prognosis in the healthy population and in patients with structural heart disease (7–9).

PA data can be collected constantly and automatically using accelerometer sensors (10–12). Previous studies evaluating PA, when used alone or when integrated with other diagnostic procedures in patients with ICD/CRT-D, suggested an inverse relationship with HF regarding hospitalization and survival, after adjusting for other clinical factors (6, 13–17). PA level in males is different from that in females in various diseases (18–20). Despite the important role of PA in the prognostication of outcomes after ICD/CRT-D implantation, little is known about the differences between sex in PA levels among those patients.

The present study aimed to clarify the impact of sex on PA levels among patients with ICD/CRT-D and their predictive value for cardiac death and all-cause mortality risk.

## Methods

### Study Population and Study Design

We retrospectively analyzed patients who underwent ICD or CRT-D implantation between June 2010 and June 2014 from the SUMMIT registry study. The present study complied with the Declaration of Helsinki, and ethics committees from all participating institutions approved this study. All patients provided written informed consent before enrollment. Inclusion criteria: (1) patients with indications for ICD/CRT-D (Biotronik SE & Co. KG; Berlin, Germany) implantation according to Class I therapy practice guidelines; (2) patients with ICD/CRT-D devices equipped to process daily home monitoring (HM) transmissions; (3) patients aged over 18 years; (4) patients who survived more than three months after ICD/CRT-D implantation. Exclusion criteria: (1) unable to follow up as required or had missing HM data; (2) with diagnosed malignant tumor or life expectancy <1 year; (3) scheduled heart transplant. Eight hundred and forty-five patients from a total of 1,008 patients had PA data recorded. Nineteen patients with incomplete data, 3 patients who died within three months after implantation, 3 patients lost to follow-up were excluded, and 820 patients were eventually enrolled.

### Baseline Clinical Characteristics

Information regarding baseline clinical characteristics was collected from patient medical records before ICD/CRT-D implantation, which included age, sex, and body mass index (BMI), New York Heart Association (NYHA) class, ischemic cardiomyopathy (ICM) history, old myocardial infarction and valvular disease history, comorbidities [hypertension, diabetes, atrial fibrillation (AF), stroke and pre-implantation syncope], echocardiographic characteristics [left ventricular ejection fraction (LVEF), and left ventricular end-diastolic diameter (LVEDD)], and medication history [beta-blockers, amiodarone, diuretics, statins, digoxin, aldosterone antagonist, and angiotensin-converting enzyme inhibitors (ACEI) or angiotensin receptor blockers (ARB)].

### PA Measurement

PA in ICD/CRT-D was measured through an integrated circuit accelerometer embedded in the pulse generator. The accelerometer detects both the frequency and amplitude of patient motion and translates this into a proportional electrical signal updated each minute. Automatic PA measurements using Biotronik accelerometer sensors were cumulatively collected and recorded as the time when the device delivered higher than the devices' basic rates. The PA resolution was 2 s, and the data were converted into % per 24 h. The Biotronik remote monitoring system can automatically transmit data stored in implantable devices to the Biotronik service center every day. Considering clinical procedural recovery, the baseline PA was defined as the average PA measured from 30 to 60 days after device implantation, as recommended by previous studies (14–16).

### Follow-Up and Study Endpoints

Patients were immediately informed in case of interruption (failure or disruption) in data transmission. Routine follow-up clinic visits and phone calls were conducted by designated clinical coordinators. The primary endpoint of this study was cardiac death. The secondary endpoint was all-cause mortality. In case of patient death, the date and cause of death was confirmed with their families based on the death certificate.

### Statistical Analysis

Continuous variables are expressed as mean ± standard deviations (SD), and were compared between the male group and female group using Student's *t*-test. Categorical variables are expressed as numbers and percentages and were compared between the two groups using Pearson's χ^2^ test. Receiver operating characteristic (ROC) curves were plotted to identify a PA cut-off value in the male group and female group, which was used to predict cardiac death and all-cause mortality. Kaplan–Meier survival curves (Log-rank tests) evaluated survival time from the date of ICD or CRT-D implantation to the date of cardiac death and all-cause mortality between subgroups of PA<cutoff AND PA ≥cut-off in males and females, respectively. Univariate and multivariate Cox regression analyses were used to evaluate the relationship among the baseline characteristics, which included the PA level and the study endpoints. Hazard ratios and 95% confidence intervals (CIs) were calculated for the endpoints. Based on the two PA cut-offs for the male and female groups, the participants were categorized into three subgroups. The endpoints were compared between males and females when PA was between the cut-off values of males and females. All statistical analyses were performed using SPSS (IBM SPSS Statistics for Windows, Version 23.0. Armonk, NY: IBM Corp.) and Graph Pad Prism Software (version 5.0; GraphPad Software, La Jolla, CA, USA). Two-sided statistical significance was defined as *P* value < 0.05 for all analyses.

## Results

### Baseline Characteristics

Baseline demographic and clinical characteristics are shown in Table 1. Overall, 820 patients, of which 73.9% were males (*n* = 606), were enrolled in this study. Their mean age was 60.84 ± 13.85 years. The mean LVEF and LVEDD were 42.70 ± 14.95% and 58.53 ± 13.08 mm, respectively, with 26.2% of patients receiving CRT-D devices. The mean baseline PA level was significantly higher in the male group than in the female group (11.40 ± 5.83% vs. 9.93 ± 5.49%, *P* = 0.001). Compared to patients in the female group, those in the male group had higher BMI (*P* < 0.001) and LVEDD (*P* < 0.001), more concomitant with ICM (*P* = 0.015) and received statin (*P* = 0.024) and amiodarone therapy (*P* = 0.031).

**Table 1:** Baseline characteristics.

**Variables**	**Total *n* = 820**	**Male *n* = 606**	**Female *n* = 214**	***P* value**
Age at implantation, years	60.84 ± 13.85	60.66 ± 13.75	61.34 ± 14.14	0.536
BMI, kg/m2	23.62 ± 3.53	23.93 ± 3.48	22.75 ± 3.54	<0.001
PA, %	11.01 ± 5.78	11.40 ± 5.83	9.93 ± 5.49	0.001
Primary prevention	433 (52.8%)	322 (53.1%)	111 (51.9%)	0.751
NYHA, class I–II	421 (51.3%)	315 (52%)	106 (49.5%)	0.578
CRT-D	215 (26.2%)	156 (25.7%)	59 (27.6%)	0.651
Comorbidities				
ICM	282 (34.4%)	383 (36.8%)	59 (27.6%)	0.015
Hypertension	259 (31.6%)	194 (32.0%)	65 (30.4%)	0.670
Diabetes	78 (9.5%)	65 (10.6%)	14 (6.5%)	0.103
Stroke	16 (2%)	9 (1.5%)	7 (3.3%)	0.146
AF	90 (11.0%)	62 (10.2%)	28 (13.1%)	0.254
Valvular disease	20 (2.4%)	12 (2.0%)	8 (3.7%)	0.194
Old myocardial infarction	127 (15.5%)	110 (18.2%)	17 (7.9%)	<0.001
Pre-implant syncope	174 (21.8%)	132 (21.8%)	42 (19.6%)	0.560
Echocardiography				
LVEF, %	42.70 ± 14.95	42.42 ± 15.03	43.50 ± 14.73	0.362
LVEDD, mm	58.53 ± 13.08	59.64 ± 13.26	55.40 ± 12.03	<0.001
Medication				
Beta-blockers	505 (61.6%)	368 (60.7%)	137 (64.0%)	0.414
ACEI or ARB	319 (38.9%)	226 (37.3%)	93 (43.5%)	0.121
Aldosterone antagonist	293 (35.7%)	217 (35.8%)	76 (35.5%)	1.000
Statins	192 (23.4%)	154 (25.4%)	38 (17.8%)	0.024
Diuretic	338 (41.2%)	251 (41.4%)	87 (40.7%)	0.845
Digoxins	170 (20.7%)	124 (20.5%)	46 (21.5%)	0.759
Amiodarone	247 (30.1%)	195 (32.2%)	52 (24.3%)	0.031

*ACEIs, angiotensin-converting enzyme inhibitors; ARBs, angiotensin receptor blockers; BMI, Body Mass Index; CRTD, cardiac resynchronization therapy and implantable cardioverter-defibrillator; ICM, ischemic cardiomyopathy; LVEDD, left ventricular end -diastolic dimension; LVEF, left ventricular ejection fraction; NYHA class, New York Heart Association class; PA, physical activity*.

### Clinical Outcomes and Baseline PA Level as a Predictor

During a mean follow-up time of 75.7 ± 29.1 months (range from 3.1 to 116.6 months), 90 cardiac (11.0%) and 181 all-cause mortality events (22.1%) occurred.

**Figure 1:**
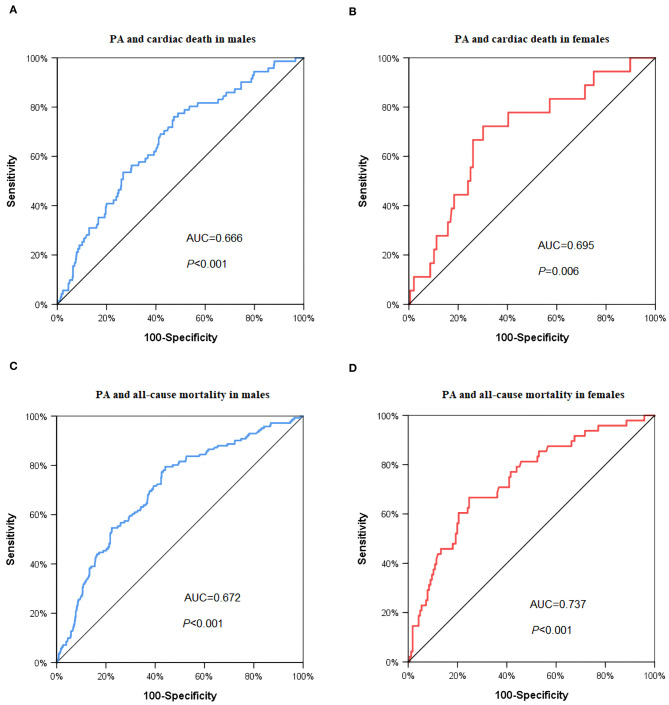
ROC curve to predict the cut-off values of cardiac death and all-cause mortality. **(A)** Cardiac death in male group: AUC = 0.666, *P* < 0.001, PA cut off value = 11.16%; **(B)** cardiac death in female group: AUC = 0.695, *P* = 0.006, PA cut-off value = 7.15%; **(C)** all-cause mortality in male group: AUC = 0.702, *P* < 0.001, PA cut off value = 11.33%; **(D)** all-cause mortality in female group: AUC = 0.737, *P* < 0.001, PA cut off value = 7.17%. PA: physical activity.

In the ROC analysis of baseline PA levels as a predictor for cardiac death (Figure 1), the cut-off value was 11.16% in the male group (area under the curve (AUC) = 0.666, *P* < 0.001, Figure 1A) and 7.15% in the female group (AUC = 0.695, *P* = 0.006, Figure 1B), respectively. The cut-of value of baseline PA levels as a predictor for all-cause mortality was 11.33% in the male group (AUC = 0.702, *P* < 0.001, Figure 1C), and 7.17% in the female group (AUC = 0.737, *P* < 0.001), respectively (Figure 1D).

### Kaplan–Meier Survival Curves

**Figure 2:**
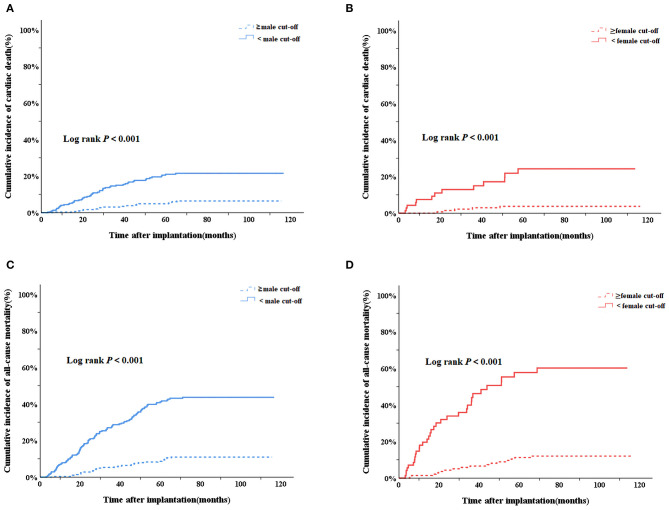
Kaplan–Meier survival curves to evaluate the cumulative incidence of cardiac death and all-cause mortality between subgroups of PA<cutoff AND PA ≥cut-off in males and females, respectively. **(A)** Cardiac death in male group; **(B)** cardiac death in female group; **(C)** all-cause mortality in male group; **(D)** all-cause mortality in female group. PA, physical activity.

As shown in Figure 2, in the Kaplan-Meier survival analysis, patients with baseline PA levels lower than cut-off values had higher cumulative incidence of cardiac death in both males (Log rank *P* < 0.001, Figure 2A) and females (Log rank *P* < 0.001, Figure 2B). The results were similar regarding all-cause mortality. With baseline PA levels lower than cut-off values, males (Log rank *P* < 0.001, Figure 2C) and females (Log rank *P* < 0.001, Figure 2D) both had higher cumulative incidence of all-cause mortality.

### Cox Regression Analysis in Males and Females

As shown in Table 2, uni-variate and multivariate cox regression analysis were performed in males and females, respectively. In the males group, the uni-variate Cox regression model showed that PA levels <11.16% were associated with an increased risk of cardiac death (HR = 3.460; 95%CI = 2.029–5.902, *P* < 0.001) and PA levels <11.33% were associated with an increased all-cause mortality (HR = 4.065; 95%CI = 2.716–6.084, *P* < 0.001). Both PA measurements (<11.16% and <11.33%) remained as independent predictors of cardiac death (HR = 3.071, 95%CI = 1.741–5.417, *P* < 0.001) and all-cause mortality (HR = 3.445, 95%CI = 2.246–5.283, *P* < 0.001), when adjusted using a multivariate model (adjusted for age, NYHA class, BMI, primary indication, CRT-D, hypertension, diabetes, AF, stroke, ICM, LVEF, LVEDD, old myocardial infarction, beta-blockers, aldosterone antagonist, ACEI or ARB, diuretics, amiodarone and statins).

**Table 2:** Predictors of cardiac death and all-cause mortality according to PA in male and female patients.

	**Uni-variate HR (95% CI)**	***P* value**	**Multivariate HR (95% CI)**	***P* value**
**MALE**				
Cardiac death PA category, PA <11.16%	3.460 (2.029-5.902)	<0.001	3.071 (1.741-5.417)	<0.001
All-cause mortality PA category, PA <11.33%	4.065 (2.716-6.084)	<0.001	3.445 (2.246-5.283)	<0.001
**FEMALE**				
Cardiac death PA category, PA <7.15%	6.583 (2.343-18.499)	<0.001	9.846 (2.636-36.782)	0.001
All-cause mortality PA category, PA <7.17%	5.188 (2.852-9.438)	<0.001	4.510 (2.355-8.638)	<0.001

*CI, confidence interval; HR, hazard ratio; PA, physical activity*.

In the female group, PA levels <7.15% were associated with an increased risk of cardiac death (HR = 6.583; 95%CI = 2.343–18.499, *P* < 0.001) and PA levels <7.17% were associated with an increased all-cause mortality (HR = 5.188; 95%CI = 2.852– 9.438, *P* < 0.001) in a uni-variate Cox regression model. PA levels <7.15% and <7.17% remained as independent predictors of cardiac death (HR = 9.846, 95%CI = 2.636–36.782, *P* = 0.001) and all-cause mortality (HR = 4.510, 95%CI = 2.355–8.638, *P* < 0.001), when adjusted in a multivariate model (adjusted for age, NYHA class, BMI, primary indication, CRT-D, hypertension, diabetes, AF, stroke, ICM, LVEF, LVEDD, old myocardial infarction, beta-blockers, aldosterone antagonist, ACEI or ARB, diuretics, amiodarone and statins).

### Sex Differences in the Subgroup of PA Levels Between the Cut-Off Values of Males and Females

As shown in Table 3, in the subgroup of PA levels between the cut-off values of males and females, males had a higher risk of cardiac death (hazard ratio = 4.952; 95%CI = 1.055-23.245, *P* = 0.043) and all-cause mortality (hazard ratio = 2.432; 95% CI = 1.095−5.402, *P* = 0.029) after adjusting for confounders (age, PA, NYHA class, BMI, primary indication, CRT-D, hypertension, diabetes, AF, stroke, ICM, LVEF, LVEDD, old myocardial infarction, betablockers, aldosterone antagonist, ACEI or ARB, diuretics, amiodarone and statins).

**Table 3:** Sex differences in the subgroup of PA levels between the cut-off values of males and females.

	**Uni-variate HR (95% CI)**	***P* value**	**Multivariate HR (95% CI)**	***P* value**
Cardiac death	4.735 1.119-20.038)	0.035	4.952 1.055-23.245)	0.043
All-cause mortality	2.061 1.011-4.201)	0.047	2.432 1.095-5.402)	0.029

*CI, confidence interval; HR, hazard ratio*.

## Discussion

### Main Findings

Our main findings were as follows: (1) patients with baseline PA levels lower than cut-off values had higher cumulative incidence of cardiac death and all-cause mortality in both males and females; (2) male patients have higher baseline and predictive PA cut-off values than female patients; and (3) the difference between males and females was observed when the PA level was between the cut-off values of males and females.

### Relationship Between PA and Outcomes

A series of studies evaluated the relationship between daily PA levels and outcomes in ICD/CRT-D patients, including hospitalization due to HF, cardiac death, and all-cause mortality (6, 13–17). Previous studies discovered that an integrated diagnostic algorithm derived from device-measured dynamic parameters, including PA, could identify CRT-D patients with a higher risk of hospitalization due to HF within the subsequent months (6, 13). Conraads et al. demonstrated that the PA during the early 30-day period post device implantation as an independent significant predictor of hospitalization due to HF and all-cause mortality even after correcting for HF severity (14). Palmisano et al. provided more evidence about the relationship between PA and hospitalization due to HF, and Kramer et al. discovered that regardless of baseline and time-varying PA levels, a low PA level was associated with a higher risk of all-cause mortality (15, 17). Zhao et al. demonstrated that a PA cut-off value of 7.84% can predict the risk of cardiac death and all-cause mortality in patients with ICD/CRT-D implantation (16). In the present study, significant clinical factors such as age, NYHA class, LVEF, CRT-D, comorbidities and medications were taken into consideration in the multivariate Cox regression analysis, and more evidence was provided to support the inverse association between daily PA and cardiac death and all-cause mortality. Additionally, PA was recorded with home monitoring and the association was accessed in males and females, respectively.

### Sex Differences in PA and Its Effects on Endpoints Risk

Previous studies have examined sex differences in PA levels after a cardiac event (18–20). The present study showed conclusive evidence that PA levels were higher in males than in females among patients with ICD/CRT-D. In addition, the PA cut-off value predicting negative outcomes was higher in male patients than in female patients. When the PA level was between the cut-off values of PA in males and females, male patients had a 5-fold increased risk for cardiac death and 2.5-fold increased risk for all-cause mortality, compared with female patients. Therefore, female sex may be a protective factor in patients with insufficient PA. Our results were consistent with those of previous studies suggesting that females may have a lower risk of exertion-related sudden cardiac death (21–23). Although specific underlying mechanisms have not been elucidated, possible explanations may be due to the important role of the autonomic nervous system in cardiac death. Estrogen, a predominant hormone in females, modulates the autonomic nervous system and increases vagal tone (24). In a study conducted by Zhao et al. a high PA level was an indicator of efficient autonomic function because of a significant correlation between PA and heart rate variability (16). Thus, both high PA levels and female sex contributed to a better prognosis in patients with ICD/CRT-D by modulating autonomic function.

### Clinical Implications

Male patients were shown to present with more severe disease, have more comorbidities, and receive more medications than females. Male patients were also shown to engage in more PA after ICD/CRT-D implantation than female patients. Female patients were shown to have greater benefits from increased PA. Sex differences were shown to impact PA levels, cardiac death, and all-cause mortality of patients who received ICD/CRT-D. Thus, interventions to improve the outcomes in patients with ICD/CRT-D must consider sex as an important contributing factor.

## Limitations

The present study was a retrospective study. Of the 820 patients recruited, only 214 female patients (26.1%), compared to the 606 (73.9%) male patients, were included, which may lead to selection bias. In addition, the present study included a mixture of patients receiving ICDs or CRT-Ds for primary or secondary prevention. Moreover, despite of adjustment for multiple covariates, some important basal biochemical and clinical characteristics, like the levels of glucose and lipid profile, smoke or alcohol intake, were not included. And the specific underlying mechanisms explaining the impact of sex on PA levels and their association with outcomes were not elucidated, although we attempted to provide possible explanations. Thus, more prospective studies with larger samples are needed to further validate our findings.

## Conclusions

Baseline PA levels are significantly higher in male patients than in female patients with ICD/CRT-D. Male patients have higher predictive PA cut-off values for cardiac death and all-cause mortality than female patients. The present study suggests that sex should be considered as an important contributing factor when deciding for PA targets in patients with ICD/CRT-D.

## Data Availability Statement

The raw data supporting the conclusions of this article will be made available by the authors, without undue reservation.

## Ethics Statement

The studies involving human participants were reviewed and approved by ethics committee of Fuwai Hospital (the chief institute) and all other participating organizations (Zhongshan Hospital Fudan University et al.). The patients/participants provided their written informed consent to participate in this study.

## Author Contributions

XL and SZhang contributed to the conception or design of the work. XL, ZLi, and SZhao contributed to the acquisition, analysis, and interpretation of data for the work. XL, XX, and XS drafted the manuscript. KC, WH, YS, JY, ZLia, WX, and SZhang critically revised the manuscript. All authors gave final approval and agree to be accountable for all aspects of work ensuring integrity and accuracy.

## Conflict of Interest

The authors declare that the research was conducted in the absence of any commercial or financial relationships that could be construed as a potential conflict of interest.

## Abbreviations

PA: physical activity
SCD: sudden cardiac death
CIED: cardiovascular implantable electronic devices
HM: home monitoring
ICD: implantable cardioverter-defibrillator
CRTD: cardiac resynchronization therapy defibrillator
AF: atrial fibrillation
ACEIs: angiotensin-converting enzyme inhibitors
ARBs: angiotensin receptor blockers
BMI: body mass index
LVEDD: left ventricular end diastolic dimension
LVEF: left ventricular ejection fraction
NYHA: New York Heart Association
HR: hazard ratio
CI: confidence interval.
